# SARS‐CoV‐2 B.1.1.7 reinfection after previous COVID‐19 in two immunocompetent Italian patients

**DOI:** 10.1002/jmv.27066

**Published:** 2021-05-15

**Authors:** Federica Novazzi, Andreina Baj, Angelo Genoni, Pietro G. Spezia, Alberto Colombo, Gianluca Cassani, Cristian Zago, Renee Pasciuta, Daniela Della Gasperina, Walter Ageno, Paolo Severgnini, Francesco Dentali, Daniele Focosi, Fabrizio Maggi

**Affiliations:** ^1^ Laboratory of Microbiology ASST Sette Laghi Varese Italy; ^2^ Department of Medicine and Surgery University of Insubria Varese Italy; ^3^ Department of Translational Research University of Pisa Pisa Italy; ^4^ Unit of Internal Medicine ASST dei Sette Laghi Varese Italy; ^5^ Department of Emergency ASST dei Sette Laghi Varese Italy; ^6^ Biotechnology and Life Sciences Department, Anesthesia and Intensive Care ASST Sette Laghi Varese Italy; ^7^ North‐Western Tuscany Blood Bank Pisa University Hospital Pisa Italy

**Keywords:** 20I/501Y.V1, B.1.1.7, COVID‐19, SARS‐CoV‐2, UK variant, variant of concern, VOC 202012/01

To date, only one case of SARS‐COV‐2 B.1.1.7 reinfection has been reported.^1^ We report here two more such reinfection cases in Lombardy residents that, nevertheless the ECDC statement of a period from 45 to 90 days to confirm reinfection,^2^ experimented a second infection from a B.1.1.7 variant of SARS‐CoV‐2 only 1 month after the first one. In both cases, interstitial pneumonia requiring intubation or oxygen support was present at the time of the first infection, whereas the second one was characterized by very mild development.

Case 1 was a 56‐year‐old immunocompetent male, a former smoker with obesity and dyslipidemia. He was employed as a truck driver, moving across Switzerland, Austria, and Germany. On December 31, 2020, he presented at Varese hospital's emergency room with moderate dyspnea; he was discharged and treated at home with levofloxacin, corticosteroids, and low molecular weight heparin. Clinical conditions worsened, and he was readmitted to the hospital on January 3, when interstitial pneumonia was diagnosed and treated with continuous positive airway pressure (CPAP) ventilation was initiated. On January 4, he tested positive for SARS‐CoV‐2 RNA on the nasopharyngeal swab (NPS) (1242 RLU on Hologic Panther; *C*_t_ 10 on Abbott m2000). On January 6, he was moved to the intensive care unit (ICU) and intubated. On January 10, the patient was moved to the ICU of Milan hospital for logistical reasons, pronated, and finally extubated on January 20. He was then moved to the COVID ward: the radiological pattern of pneumonia showed marked improvement, and NPSs tested negative for SARS‐CoV‐2 RNA on January 23, January 31, and February 2. On February 3, he was moved to a different hospital for rehabilitation. On February 4, a new NPS‐tested positive for SARS‐CoV‐2 RNA (1233 RLU on Hologic Panther; *C*_t_ 24 on Abbott m2000), while anti‐SARS‐CoV‐2 immunoglobulin G (IgG) was 194 AU/ml (LIAISON® SARS‐CoV‐2 Ab; DiaSorin), and C‐reactive protein rose to 64 mg/L, but without clinical worsening. On February 5, serology was repeated on a new sample with the concordant result (169 AU/ml), but additional NPSs tested negative for SARS‐CoV‐2 RNA on February 5, February 6, and February 11. At this point, we confirm the real‐time polymerase chain reaction (PCR) results testing a new aliquot of the positive NPSs starting from an independent extraction and we sequenced twice the RBD fragment of the spike gene from different aliquots of each NPS sample dated January 4 and February 4, as previously reported.^3^ While the January 4, S region showed no mutations when compared with SARS‐CoV‐2 isolate Wuhan‐Hu‐1 (deposited in GenBank as MW599237), the February 4 strain resulted in B.1.1.7, with aminoacid substitution N501Y and A570D (deposited in GenBank as MW599860).

Case 2 was a 58‐year‐old immunocompetent male who tested positive for SARS‐CoV‐2 RNA on NPS on January 7, 2021. He was treated at home with azithromycin, enoxaparin, and prednisone, which required hospital admission on January 18, where interstitial pneumonia was diagnosed. On January 19, he tested positive for SARS‐CoV‐2 RNA on NPS (1302 RLU on Hologic Panther), and he was moved to the COVID ward for oxygen support, progressing to CPAP ventilation on January 21. CPAP was discontinued on January 26, and two follow‐up NPSs on January 31, and February 2 were negative for SARS‐CoV‐2 RNA. New NPSs on February 4 and 6 were positive for SARS‐CoV‐2 RNA (1181 RLU on Hologic Panther, and *C*_t_ 27 on m2000 Abbott, respectively), with anti‐RBD IgG > 400 AU/ml, and without clinical worsening. On February 10 the patient was moved to the subacute medical unit. At that point, we confirm the real‐time PCR results for the above patient and we decided to sequence twice the RBD from aliquots of NPS samples dated January 19 and February 6: the January 19 S sequence showed absence of mutation if compared with SARS‐CoV‐2 isolate Wuhan‐Hu‐1 (deposited in GenBank as MW599251), while the February 6 strain resulted in B.1.1.7, with aminoacid substitution N501Y and A570D (deposited in GenBank as MW599954).

According to Facebook mobility data,^4^ in 16 of 19 countries analyzed, there is at least a 50% chance the variant was already imported by travelers from the United Kingdom by December 7,^5^ with Italy being the country with the highest risk. Accordingly, many cases have been reported in Lombardy. Theoretical models have estimated the reinfection rate at 0.7%, similar to older strains,^6^ so that many more cases are likely undetected.

## CONFLICT OF INTERESTS

The authors declare that there are no conflict of interests.

## AUTHOR CONTRIBUTIONS

Federica Novazzi, Andreina Baj, Daniele Focosi, and Fabrizio Maggi contributed to study design, data collection, data interpretation, the literature search, and writing of the paper. Daniela Della Gasperina, Walter Ageno, Paolo Severgnini, and Francesco Dentali contributed to patients' recruitment, data collection, and clinical management. Angelo Genoni, Pietro G. Spezia, Alberto Colombo, Gianluca Cassani, Cristian Zago, and Renee Pasciuta managed the laboratory diagnostic procedures and collected data. Fabrizio Maggi supervised the clinical activity of the project. All authors reviewed and approved the final version of the report.
